# The Role of the AggR Regulon in the Virulence of the Shiga Toxin-Producing Enteroaggregative *Escherichia coli* Epidemic O104:H4 Strain in Mice

**DOI:** 10.3389/fmicb.2019.01824

**Published:** 2019-08-13

**Authors:** Nadia Boisen, Angela R. Melton-Celsa, Anne-Marie Hansen, Tonia Zangari, Mark A. Smith, Lisa M. Russo, Flemming Scheutz, Alison D. O’Brien, James P. Nataro

**Affiliations:** ^1^Department of Bacteria, Parasites and Fungi, Statens Serum Institut, Copenhagen, Denmark; ^2^Department of Pediatrics, University of Virginia School of Medicine, Charlottesville, VA, United States; ^3^Department of Microbiology and Immunology, Uniformed Services University of the Health Sciences, Bethesda, MD, United States; ^4^Department of Microbiology and Immunology, University of Maryland School of Medicine, Baltimore, MD, United States

**Keywords:** enteroaggregative *Escherichia coli*, O104:H4, Shiga toxin, diarrhea, mouse model

## Abstract

An O104:H4 Shiga toxin (Stx)-producing enteroaggregative *Escherichia coli* (EAEC) strain caused a large outbreak of bloody diarrhea and the hemolytic uremic syndrome in 2011. We previously developed an ampicillin (Amp)-treated C57BL/6 mouse model to measure morbidity (weight loss) and mortality of mice orally infected with the prototype Stx-EAEC strain C227-11. Here, we hypothesized that mice fed C227-11 cured of the pAA plasmid or deleted for individual genes on that plasmid would display reduced virulence compared to animals given the wild-type (wt) strain. C227-11 cured of the pAA plasmid or deleted for the known pAA-encoded virulence genes *aggR*, *aggA*, *sepA*, or *aar* were fed to Amp-treated C57BL/6 mice at doses of 10^10^–10^11^CFU. Infected animals were then either monitored for morbidity and lethality for 28 days or euthanized to determine intestinal pathology and colonization levels at selected times. The pAA-cured, *aggR*, and *aggA* mutants of strain C227-11 all showed reduced colonization at various intestinal sites. However, the *aggR* mutant was the only mutant attenuated for virulence as it showed both reduced morbidity and mortality. The *aar* mutant showed increased expression of the aggregative adherence fimbriae (AAF) and caused greater systemic effects in infected mice when compared to the C227-11 wt strain. However, unexpectedly, both the *aggA* and *aar* mutants displayed increased weight loss compared to wt. The *sepA* mutant did not exhibit altered morbidity or mortality in the Amp-treated mouse model compared to wt. Our data suggest that the increased morbidity due to the *aar* mutant could possibly be via an effect on expression of an as yet unknown virulence-associated factor under AggR control.

## Introduction

Enteroaggregative *Escherichia coli* (EAEC) are members of a diarrheagenic pathotype linked to acute and persistent diarrhea in many settings (Bhan et al., 1989; Fang et al., 1995; Wanke et al., 1998; Okeke et al., 2000b; Adachi et al., 2001; Valentiner-Branth et al., 2003; Sahl et al., 2011). EAEC strains express several virulence factors (Okeke et al., 2000a; Jenkins et al., 2005; Boisen et al., 2009, 2012; Lima et al., 2013) encoded on the chromosome or on the EAEC-specific pAA plasmid. Most EAEC strains harbor a transcriptional activator called AggR (Nataro et al., 1994) that activates several factors encoded on the pAA plasmid and on the chromosome. Recently we described a novel protein negative regulator termed Aar (AggR-activated regulator) that is activated by AggR but then down-regulates expression of AggR itself and thereby the AggR regulon (Santiago et al., 2014). The genes under AggR control include those that encode the Aggregative Adherence Fimbriae (AAF), of which at least five variants exist (Nataro et al., 1993; Czeczulin et al., 1997; Henderson et al., 1999; Bernier et al., 2002; Boisen et al., 2008; Jønsson et al., 2015). AAFs are necessary for adherence to human intestinal explants and elicit both cytokine release and opening of epithelial cell tight junctions (Strauman et al., 2010).

In Germany in 2011 an EAEC strain of the serotype O104:H4 that was lysogenized with a Stx2a-converting phage caused an outbreak of foodborne hemorrhagic colitis (Scheutz et al., 2011). Over 4000 individuals were affected, and the hemolytic uremic syndrome (HUS) developed in 22% of the cases with 54 deaths (Frank et al., 2011). Analysis of the O104:H4 strain revealed numerous EAEC virulence genes, including those required for expression and function of the AAF/I variant, as well as three serine protease autotransporter proteins (SPATEs), Pic, SigA and SepA.

We have shown that deletion of *aggR* or *aggA* in the outbreak prototype strain C227-11 significantly reduced bacterial adherence and, independently, translocation of Stx2a across the T84 cell monolayer (Boisen et al., 2014). Moreover, deletion of *aggR*, *aggA*, *sepA*, or the Stx2a-encoding phage from C227-11 resulted in reduced secretion of IL-8 from the infected monolayer (Boisen et al., 2014). These latter results suggest that the AggR-regulated AAF/I fimbriae enhance inflammation and enable the outbreak strain to adhere to epithelial cells and foster Stx2a translocation across the intestinal epithelium.

Furthermore, we have reported that ampicillin (Amp)-treated C57BL/6 mice infected with C227-11 lost weight, developed colitis and renal damage over the course of infection and a portion of the animals died (Zangari et al., 2013). Mice infected with a Stx negative C227-11 strain showed significantly reduced morbidity and no mortality (Zangari et al., 2013), findings that indicate that death in that animal model is primarily due to Stx2a. Here, we investigate the role in virulence of four EAEC genes, *aggR*, *aggA*, *aar*, and *sepA* that are encoded on the pAA plasmid in the Amp-treated mouse model (Zangari et al., 2013).

## Materials and Methods

### Bacterial Strains, Plasmids, and Growth Conditions

Bacterial strains and plasmids used in this study are described in Table 1. Strains were grown at 37°C in Luria-broth (LB) or Dulbecco’s Modified Eagle’s medium (DMEM) supplemented with 0.45% glucose. Recovery of bacteria from fecal pellets or tissues of mice was done on MacConkey or LB agar. When indicated, media were supplemented with kanamycin (Km) (50 μg/ml), carbenicillin (Ca) (100 μg/ml), tetracycline (Tc) (30 μg/ml), streptomycin (Str) (50 μg/ml), penicillin (Pen) (50 μg/ml), chloramphenicol (Cm), (30 μg/ml) gentamicin (Gm) (100 μg/ml), and/or 0.2% arabinose.

**TABLE 1:** Strains, constructs, and oligonucleotides used in this study.

**Strain, plasmid, or oligonucleotide**	**Genotype and/or relevant characteristics**	**References**
**Strains**		
C227-11	EAEC O104:H4 Stx2a-producing strain	Scheutz et al., 2011
C227-11ϕcu	C227-11 cured of Stx2-producing lambda-like phage	Zangari et al., 2013
C227-11pAA^–^	C227-11 cured of the pAA plasmid	Boisen et al., 2014
C227-11*aggR*	C227-11 with the insertion mutation *aggR*:*km*, Km^r^	Boisen et al., 2014
C227-11*aggA*	C227-11 with the insertion deletion Δ*aggA:km*, Km^r^	Boisen et al., 2014
C227-11*sepA*	C227-11 with the insertion deletion Δ*sepA:km*, Km^r^	Boisen et al., 2014
C227-11*aar*	C227-11 with the insertion deletion Δ*aar:km*, Km^r^	This study
C227-11ϕcu *aar*	C227-11ϕcu with the insertion deletion Δ*aar:km*, Km^r^	This work
**Plasmids**		
pCR2.1-TOPO	TOPO TA cloning vector, Km^r^ Ap^r^	Invitrogen
pACYC177	Medium-copy number cloning vector, Km^r^ Ap^r^	New England Biolabs
pACYC184	Medium-copy number cloning vector, Cm^r^ Tc^r^	New England Biolabs
pSRS1	Suicide vector derivate of pJMM112, *sacB*, Ap^r^	
pAMH317	pACYC184:*aar*, Cm^r^	This study
pAMH318	pCR2.1-TOPO: Δ*aar:km*, Km^r^	This study
pAMH337	pSRS1: Δ*aar:km*, Km^r^	This study
**Oligo name**	Oligo sequence (5′ to 3′)	
AH1056	CATCATCCAGCCAGAAAGTGAGG	
AH1057	GTCAAGTCAGCGTAATGCTCTGC	
AH1200	CGTAGTCGACAGTTCATTGACTGATAGACTGGACTTGT	
AH1201	CGTAGGATCCGGGCTGAGTTCCCTCAAAGT	
AH1203	CGTACTCGAGCCAGAAGTTCGGTACTCCAG	
AH1204	GCACTCGCGAGTAGTCAACAAAAACTGTCTAGACTGGC	
AH1205	GCACTCGCGACATCAATACCTCCCAATACAATGTTCTC	
AH1218	CACCCAGCTGTCTAAATAGGCTGATTCAAGGCATTTACG	

### Plasmid and Strain Constructions

Oligonucleotides used in this study are listed in Table 1. Phusion Flash (Fermentas, PA, United States) or EasyA (Agilent, CA, United States) high-fidelity DNA polymerase were used for PCR. A complementation plasmid that harbored *aar* expressed from its native promoter was generated by cloning a DNA fragment amplified from C227-11 genomic DNA (gDNA) with primers AH1200/AH1201 into the *Bam*HI sites of pACYC184 (pAMH337). Deletion mutants of *aar* in C227-11 and C227-11φcu were constructed as follows. DNA fragments containing *aar* ∼500-600 bp flanking sequences were amplified with primers AH1203/AH1204 from C227-11 gDNA and cloned into pCR2.1-TOPO (pENTR/D-TOPO cloning kit, Invitrogen, CA, United States) to create pAMH317. Reverse PCR was carried out on pAMH317 with primers AH1204/AH1205 to delete *aar*. Amplified DNA was digested with *Nru*I and ligated to DNA encoding a Km resistance cassette amplified from pACYC177 with primers AH1056/AH1057 to yield pAMH318. C227-11 deletion mutants were constructed by allelic exchange (Boisen et al., 2014).

### Amp-Treated Mouse Model

The Amp-treated C57BL/6 mouse model was used as previously reported (Zangari et al., 2013). All animal studies were conducted in accordance with the *Guide for the Care and Use of Laboratory Animals* (National Research Council, Committee for the Update of the Guide for the Care and Use of Laboratory Animals, 2011) approved by the Institutional Animal Care and Use Committee of the Uniformed Services University of the Health Sciences and procedures complied with the guidelines in *Biosafety in Microbiological and Biomedical Laboratories* (Centers for Disease Control and Prevention and National Institutes of Health, 2007). Mice were monitored for survival and weight change for up to 28 days post-infection (p.i.). Determination of weight change, morbidity, mortality and animal euthanasia were done as previously described (Zangari et al., 2013). A total of 157 mice were used in these studies, and the distribution of animals infected per strain was as follows: C227-11 (55), C227-11/pAA^–^ (25), C227-11*aggR* (24), C227-11*aggA* (18), C227-11*sepA* (17), and C227-11*aar* (18).

### Intestinal Colonization Sites and Levels of C227-11 and Mutants in C57BL/6 Mice

To ascertain location of colonization, the cecum, large intestine, and a span of the terminal section of the small intestine equal in length to the large intestine were removed from each animal; the luminal contents were then separated from each organ by application of gentle pressure to the organ and bacterial were quantitated by colony count.

For fecal collections mice were transferred to an individual cage with no bedding for 30–40 min and pellets were collected. Both fecal pellets and tissue were weighed, and EMEM 1:10 w/v was added and homogenized. Supernatants were serially diluted, plated on LB-Amp/Kan, and incubated overnight at 37°C. Colonies were enumerated to determine the number of CFU/g sample as previously described (Zangari et al., 2013). The total level of toxicity present in each sample was calculated from the geometric mean for each group of animals. The amount of toxin present was then calculated by dividing the geometric mean CD_50_ by the specific activity of that toxin (CD_50_/ug). A total of 93 mice were used in these studies, and the number of animals infected per strain or uninfected were as follows: C227-11 (4), C227-11/pAA^–^ (4), C227-11*aggR* (4). C227-11*aggR*(pACYC*aggR*) (4) C227-11*aggA* (4) C227-11*aggA*(pBAD*aggDCBA*) (4) C227-11ϕcu (35), or C227-11ϕcu:*aar* (23) or uninfected control mice (11).

### Histopathological Analyses

Kidneys and cecum from at least one animal at each time point in each group were removed and fixed in 10% neutral buffered formalin (Thermo Fisher Scientific L.L.C., Kalamazoo, MI, United States). Analyses were done by scoring sections. Cecal specimens were scored as follows: (0) no significant lesions, (1) minimal epithelial necrosis, (2) mild epithelial necrosis, (3) moderate epithelial necrosis, (4) marked epithelial necrosis and (5) severe epithelial necrosis. Renal pathology was scored as follows: (0) no significant pathology, (1) minimal acute tubular necrosis (ATN), (2) mild ATN, (3) moderate ATN (4) marked ATN, and (5) severe ATN. Eight mice were utilized for these experiments: C227-11 (4) and C227-11*aar* (4).

### Hematological and Kidney Function Analyses in Mouse Studies

Blood samples were collected from four mice infected with either C227-11 or C227-11*aar* on day four or five p.i. Mice were anesthetized with isoflurane and blood was collected by cardiac puncture, and serum obtained as described (Zangari et al., 2013). Serum samples were analyzed by the Uniformed Services University of the Health Sciences Diagnostic Services and Comparative Medicine Clinical Pathology Laboratory (Bethesda, MD, United States) for levels of blood urea nitrogen (BUN) and creatinine.

### Polymerase Chain Reaction (PCR)

Loss of the bacterial pAA plasmid was assessed by lack of a signal on colony PCR that targeted plasmid-encoded *aggR* or *aatA.* Absence of the *stx*_2__a_-phage was similarly confirmed with s*tx_2__a_* as the PCR target. These PCR assays were done on 225 (C227-11), 100 (C227-pAA^–^), and 30 (C227-11*aggR*) colonies isolated from infected mice; the primers used were previously described (Boisen et al., 2014).

### Epithelial Cell Infections

Bacterial adherence to human colonic T84 epithelial cells and the subsequent impact on trans-epithelial electrical resistance (TEER) were assessed as previously described (Boisen et al., 2014).

### Vero Cell Cytotoxicity Assay

Vero cell cytotoxicity assays were done as described (Gentry and Dalrymple, 1980; Schmitt et al., 1991). Further, Shiga toxin (Stx) production were measured for C227-11, C227-11/pAA^–^, C227-11*aggR*, C227 11*sepA*, C227-11*aggA*, and C227-11*aar* over the course 2 days in two separate experiments according to standard Statens Serum Institut procedures (Scheutz, 1997).

### Tissue or Biofilm Processing for Scanning Electron Microscopy (SEM)

Scanning Electron Microscopy on samples was done at the Core Imaging Facility, University of Maryland Dental School, Baltimore as previously described (Boisen et al., 2014).

### Statistical Analyses

Statistical analyses were conducted with GraphPad Prism v6.00 (GraphPad Software, CA, United States) as specified in the figure legends.

## Results

### Contribution of pAA and pAA-Encoded Genes to C227-11 Virulence

**FIGURE 1:**
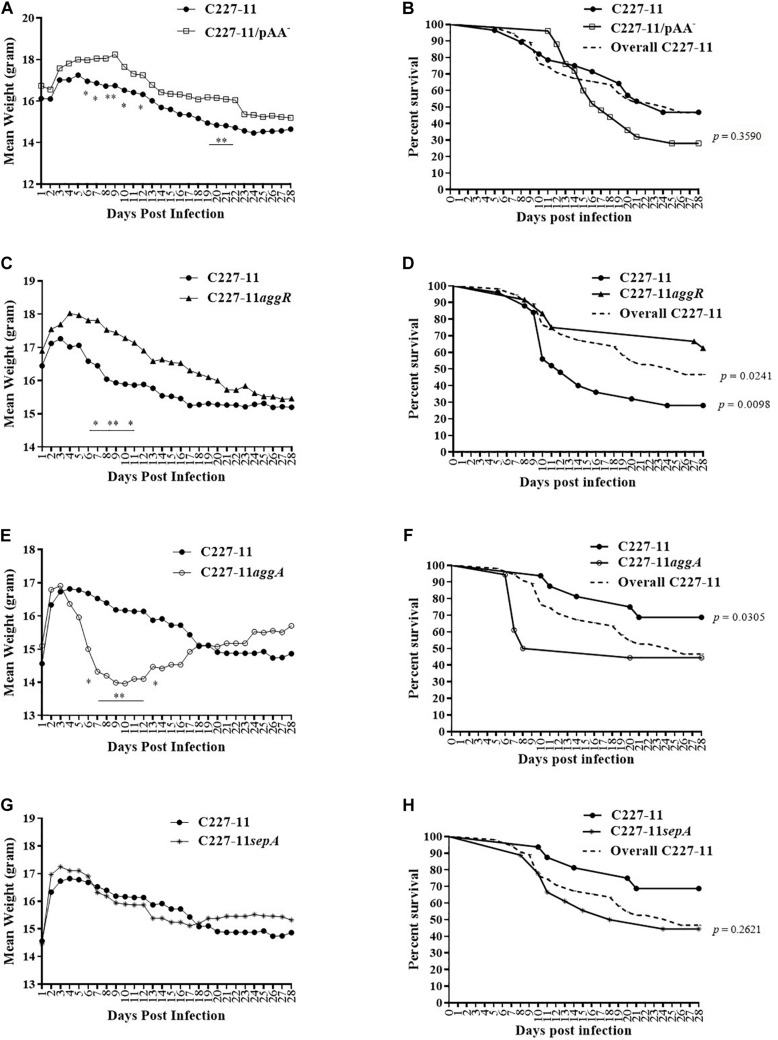
Mean weight change in grams and percent survival over time of Amp-treated C57BL/6 mice orally infected with either C227-11 (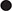) or C227-11/pAA^–^ (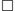) **(A,B)**, C227-11 (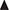) or C227-11*aggR* (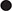) **(C,D)**, C227-11 (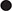) or C227-11*aggA*(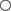) **(E,F)** or C227-11 (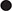) and C227-11*sepA*(^*^) **(G,H)**. Points on the graph represent the actual mean weight in grams from day one (D-1) of two or three separate experiments. Combined, 126 mice were included: 42 received C227-11, 25 C227-11/pAA^–^, 24 C227-11*aggR*, 18 C227-11*aggA*, and 17 C227-11*sepA*. The dashed line (Overall C227-11) represents all mice infected with C227-11 over the course of all animal studies included in the analysis. Differences between groups were determined by ANOVA multiple comparison with uncorrected Fisher’s least significant difference (LSD); asterisks indicate days on which a significant difference between mean weights was observed. ^*^p < 0.05 and ^∗∗^*p* < 0.005. For panels **(B,D,F,H)** differences between groups were determined by comparison of survival curves with the Log-rank (Mantel-Cox) test. The exact *p*-Values are shown on the figure.

We used the Amp-treated C57BL/6 mouse model (Zangari et al., 2013) to investigate the role of the pAA plasmid and its virulence genes in EAEC pathogenesis. We infected C57BL/6 mice with ∼10^10^ CFU of C227-11, or C227-11 cured of the pAA plasmid (C227-11/pAA^–^). Mice were monitored for 28 days for mortality and morbidity, as assessed by mean weight change from initial weight. Mice fed the C227-11 strain cured of the pAA plasmid manifested significantly less weight loss than those fed the wild type (wt) parent strain; differences reached statistical significance on days 6–10, 12, 20, and 21 p.i., and weight loss continued to be greater for wt through day 28 p.i. (Figure 1A). Mice infected with the cured pAA strain did not exhibit a mortality rate different from those fed the wt parent whether data were from wt-inoculated animals in this experiment (solid black line with circles, Figure 1B) or the aggregate of wt animal mortality from all the experiments (dashed line, Figure 1B). These latter results suggest that genes on the pAA plasmid may play a role in the morbidity of C227-11 in the Amp-treated model.

The pAA plasmid of C227-11 encodes several virulence factors that might explain the reduced weight loss in mice infected with the plasmid-cured mutant. We therefore evaluated the impact on virulence in Amp-treated mice of mutation of the gene encoding the pAA-encoded transcriptional activator AggR or other plasmid genes. A C227-11*aggR* mutant was found to exhibit a similar reduction in morbidity as was observed with the pAA-cured strain; divergence of the weight curves began on day 7 p.i. and continued for the duration of the experiment (Figure 1C), though the weight difference was statistically significant only for days 7–9 and 11. These latter results suggest that an AggR-regulated factor influences weight loss in this model. In contrast to animals fed C227-11/pAA^–^, mice fed the *aggR* mutant strain exhibited significantly lower (*p* = 0.0098) mortality than those fed the wild-type parent (Figure 1D). A statistically significant difference (*p* = 0.0241) was observed even when the total number of mice infected with C227-11 over the course of all animal studies was included in the analysis (Figure 1D, dashed line). To rule out the possibility that these observations were due to loss of *stx*_2__a_, we used PCR for *stx*_2__a_ on colonies of C227-11, C227-11pAA^–^, and C227-11*aggR* isolated from mouse feces 14 days p.i. We found that 40% of C227-11 (90/225), 22% of C227-11pAA^–^ (22/100), and 20% of C227-11*aggR* (6/30) had lost *stx*_2__a_, suggesting that loss of the Stx-encoding gene was not the likely mechanism of the mutants’ reduced virulence (data not shown). However, loss of the *stx*_2__a_-phage could have accounted for some of the variable virulence of strain C227-11 observed in the separate mouse studies.

The best characterized virulence factor encoded on the EAEC pAA plasmid is the AAF adhesin. Surprisingly, we found that a mutant in the AAF/I fimbriae (C227-11*aggA*) caused more morbidity than the wt parent. Indeed, animals infected with this mutant lost significantly more weight than the parental strain starting on day 6 p.i., before recovering (Figure 1E). In addition, mortality was significantly higher (*p* = 0.0305) for the *aggA* mutant than for the wt parent (Figure 1F). However, no difference in mortality was observed when death due to the *aggA* mutant was compared with the total number of deaths in C227-11 infected mice (Figure 1F, dashed line). This variation in C227-11 mortality between experiments in the Amp-treated C57/BL6 was noted previously (Zangari et al., 2013).

No significant differences in weight loss or overall mortality were observed in mice infected with C227-11*sepA* compared with the wt strain (Figures 1G,H, respectively).

### Colonization by C227-11 and Mutants in the Gastrointestinal Tract of C57BL/6 Mice After 24 H

**FIGURE 2:**
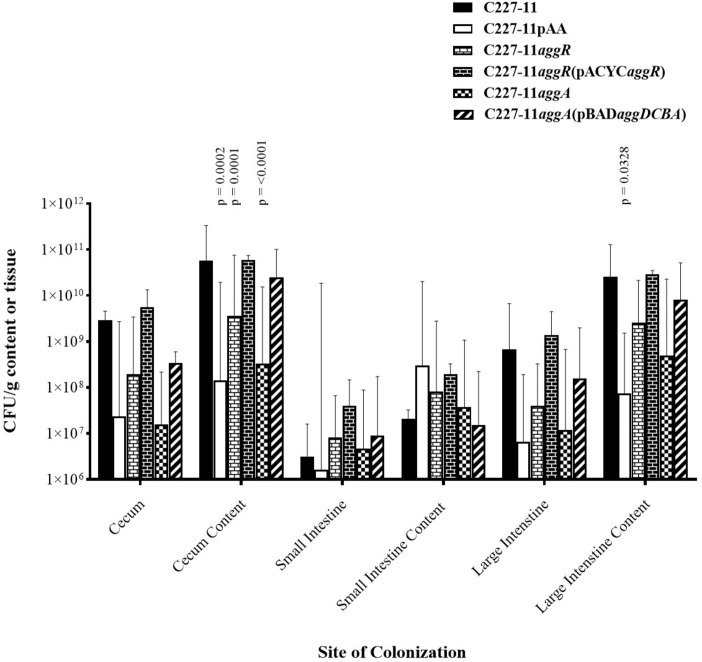
Colonization 24 h after oral infection by wild-type EAEC strain C227-11 or mutants (C227-11pAA^–^, C227-11*aggR*, C227-11*aggA*) or complemented strains [C227-11*aggR*(pACYC*aggR*), and C227-11*aggA*(pBAD*aggDCBA*)] at different sites within the gastrointestinal tract of Amp-treated C57BL/6 mice. Both the tissues (cecum, small intestine, and large intestine) and contents thereof were weighed, diluted in DMEM (1:10, wt/vol), and homogenized. Colonies from supernatants were enumerated to determine the number of CFU/g sample. Each bar represents the geometric mean with geometric mean SD from four mice. Differences between C227-11 and mutants were determined by ANOVA multiple comparison with uncorrected Fisher’s (LSD) at each site. The significant differences between mean colony count at each site were as follows; cecum content, C227-11 vs. C227-11*aggA*, *p* = < 0.0001. C227-11 vs. C227-11pAA^–^, *p* = 0.0002. C227-11 vs. C227-11*aggR*, *p* = 0.0001. Large intestine content, C227-11 vs. C227-11pAA^–^, *p* = 0.0328.

The pAA plasmid and its AAF adhesins promote adherence to human and monkey colonic tissue sections *ex vivo* (Harrington et al., 2005; Boisen et al., 2014) and in the mouse xenograft model (Boll et al., 2012). Accordingly, we quantified the abundance of C227-11, C227-11/pAA^–^, *aggR*, and *aggA* mutants and respective complemented strains at various sites in the mouse intestine (Figure 2). Groups of four mice were infected orally with either the wt or one of the three mutants and euthanized 24 h after infection. The highest levels of colonization by the wt C227-11 strain were found in cecal and colonic tissue specimens, as well as the luminal contents of those organs, as observed previously (Zangari et al., 2013; Figure 2). The organism was found in small intestinal specimens at least four orders of magnitude lower than in the cecal specimens (Figure 2).

Differences between groups were determined by ANOVA multiple comparison with uncorrected Fisher’s (LSD) on colony count values at each of the six targeted sites: cecum, cecum content, small intestines, small intestine content, large intestine, and large intestine content. After 24 h of infection, the pAA-cured mutant, as well as the *aggR*, and *aggA* mutants exhibited significantly reduced colonization, at the cecum content site compared to the wt strain (*p* = 0.0002, *p* = 0.0001, and *p* < 0.0001, respectively). This was also seen at the large intestine content site between C227-11 and C227-11pAA^–^ (*p* = 0.0328) (Figure 2). Complementation of the *aggR* and *aggA* mutants restored colonization to wt levels at all sites except the small intestine contents (Figure 2).

We measured the concentration of free Stx at each of the intestinal sites and corrected for bacterial colonization (Stx/CFU). Detectable Stx/CFU levels were lower for the wt strain than all of the other strains at all sites. These differences did not reach statistical significance (data not shown). To ascertain any differences in Stx production among the C227-11 wt strain and its mutants *in vitro*, supernatants were tested on Vero cells in two separate experiments. We found no difference in *in vitro* Stx production among the strains (data not shown).

### A Complemented *aar* Mutant of C227-11 Affects Adherence to and TEER of a T84 Cell Monolayer, or Polarized Cells, Respectively

**FIGURE 3:**
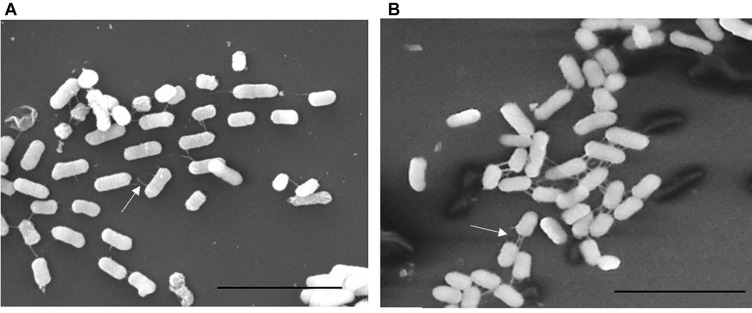
Scanning Electron Microscopy (SEM) of C227-11 **(A)** and C227-11*aar* **(B)**. White arrows indicate fimbria. Scale bar is 5 μm.

**FIGURE 4:**
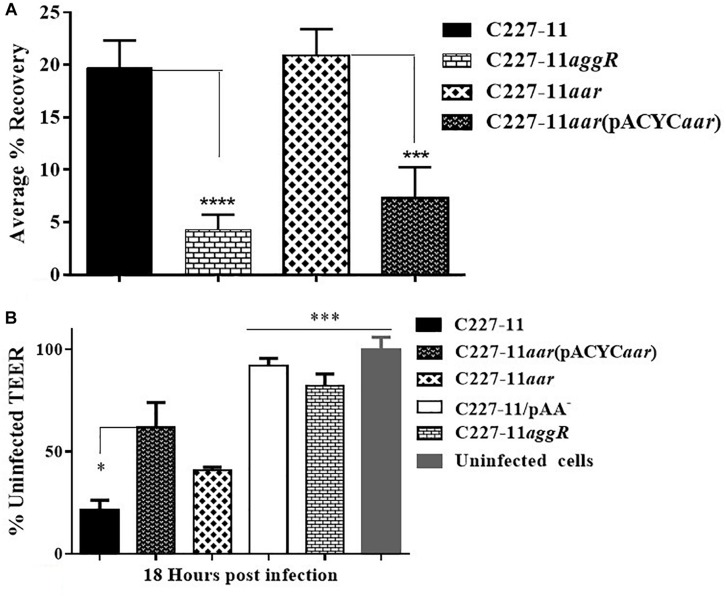
Adherence and trans-epithelial electrical resistance (TEER) of wild-type C227-11 and mutant C227-11*aar*. Adherence of C227-11 and mutants C227-11*aggR*, C227-11*aar*, and complemented strain C227-11*aar*(pACYC184*aar*) to T84 cell monolayers after 3 h incubation **(A)**. Polarized T84 cells were infected with EAEC strain C227-11, mutants C227-11/pAA^–^, C227-11*aggR*, C227-11*aar*, and C227-11*aar*(pACYC*aar*) **(B)**. 3 h after the addition of bacteria, the infection was terminated by aspiration of the apical compartment medium and addition of fresh medium containing Gm. TEER was measured 18 h after infection and reported as percent of the initial value. The data represent an average of two or three separate experiments with each done in triplicate. Error bars indicate one standard deviation of the mean adherence ^*^p < 0.05, ^∗∗^ *p* < 0.01, ^∗∗∗^*p* < 0.001 and ^*⁣*⁣**^p < 0.0001 indicate comparison between C227-11, mutants, and complemented mutants as determined by ANOVA with Tukey’s post-test.

We previously described the presence of a negative regulator (Aar) of *aggR* expression in prototype EAEC strain 042 (Santiago et al., 2014). We examined an *aar* mutant of C227-11 for fimbriation and found that the mutant displayed greater fimbriation than its parent (Figures 3A,B), as suspected from previous work. We also assessed the capacity of the C227-11*aar* to adhere to polarized T84 cells, in view of our earlier observations that the deletion of *aggA* or *aggR* significantly attenuates the capacity of C227-11 to adhere to polarized cells (Boisen et al., 2014). The *aar* mutant adhered to the surface of T84 cells to a similar degree as the wt strain; however, complementation at greater than natural copy number significantly (*p* < 0.0001) reduced adherence (Figure 4A). The reduction in adherence due to a mutation in *aggR* is shown for comparison (Figure 4A). These results support the hypothesis that Aar negatively regulates a C227-11 adhesin.

Strain C227-11 induces increased epithelial permeability in polarized T84 monolayers, as previously reported for other EAEC strains (Strauman et al., 2010; Boisen et al., 2014), and both C227-11/pAA^–^ and C227-11*aggR* induce significantly less permeability than the wt strain in the T84 cell model (Boisen et al., 2014). To characterize the effects of C227-11*aar* on intestinal barrier function, we measured the TEER of polarized infected T84 cell monolayers over 24 h from the initiation of infection. By 18 h post-infection, the C227-11*aar* mutant complemented *in trans* showed significantly (*p* < 0.5) less barrier disruption than C227-11 (Figure 4B).

### Deletion of the *aar* Gene in C227-11 Causes Significantly Increased Morbidity in C57BL/6 Mice

**FIGURE 5:**
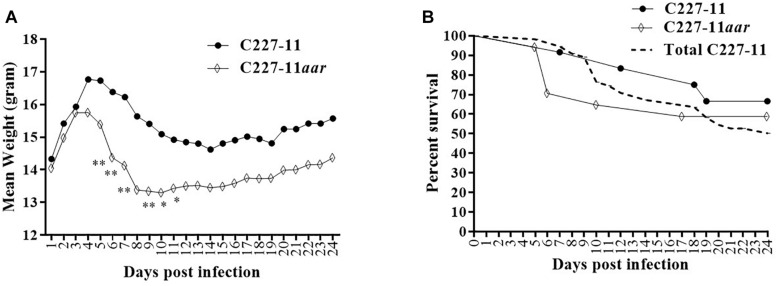
Mean weight change in gram **(A)** and percent survival **(B)** of Amp-treated C57BL/6 mice orally infected with either C227-11 (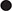) or C227-11*aar* (⋄). Points on the graph represent the mean procent weight change from day one (D-1) of two separate experiments with a total of 13 C227-11- and 18 C227-11*aar*-infected mice. The dashed line represents the combined percent survival of 55 mice infected with C227-11 (Total C227-11). Differences between groups was determined by either ANOVA with multiple comparison with uncorrected Fisher’s LSD or comparison of survival curves using Log-rank (Mantel-Cox) test. Asterisks indicate days that demonstrated a significant difference between mean weights from days post-infection. ^*^p < 0.05 and ^∗∗^*p* < 0.005.

**FIGURE 6:**
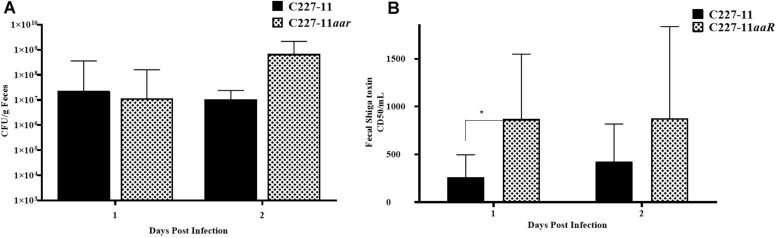
Colony forming units (CFU) per gram feces **(A)** and corresponding to Stx levels **(B)** after 1 or 2 days of infection by C227-11 and C227-11*aar* in Amp-treated C57BL/6 mice. The amount of Stx present, the 50% cytotoxicity dose (CD_50_) was determined by Vero cell assay. Each bar represents the geometric mean with geometric mean SD of eight mice. Differences between groups were determined by ANOVA multiple comparison with uncorrected Fisher’s LSD on log_10_ transformed cytotoxicity CD_50_ and colony count values; asterisks indicate sites that demonstrated a significant difference between mean colony count or Stx levels from days post-infection, ^*^p < 0.05.

The *aar* mutant demonstrated increased fimbriation by electron microscopy, yet the wt strain expressing the AAF adhesin was associated with *lower* mortality than the fimbrial mutant (C227-11*aggA*). We therefore hypothesized that the *aar* mutant (which expresses more fimbriae) would similarly demonstrate reduced morbidity and mortality. Surprisingly, the *aar* mutant caused significantly more morbidity than the wt strain (Figure 5A) although overall mortality was unchanged (Figure 5B). The *aar* mutant was shed in the stools to a similar degree as the wt strain (Figure 6A). However, fecal Stx levels were increased in mice fed the *aar* mutant on days one and two p.i. (Figure 6B), though only the difference on day 1 was significant. As with the other mutants of C227-11 we found no difference in *in vitro* Stx production among the strains when testing supernatants on Vero cells (data not shown).

### Systemic Effects of C227-11*aar* Infection

Zangari et al. (2013) concluded that Stx2a is almost certainly responsible for kidney damage and death in C227-11-infected Amp-treated mice, and that lethality could be observed as early as 5 days p.i. To assess if the systemic effects of C227-11*aar* are similar to the wt parent, we analyzed blood for hematological parameters and chemistries, as well has harvested kidneys and cecum for histopathology assessment (Table 2). Blood and organs from C227-11 and C227-11*aar*-infected mice were collected on days 4 and 5 p.i; mice were euthanized while still gaining weight on day 4 p.i and were therefore not so moribund as to impede blood collection. As we observed previously (Figure 5), the C227-11*aar*-infected mouse group showed a more rapid decline in weight on day 5 p.i. than the wt group (data not shown). The whole-blood samples were analyzed for complete blood count (CBC), and sera were evaluated for markers of kidney function, specifically, BUN, and creatinine levels (Table 2). There were no differences in the CBC between the groups on either days (data not shown). On day 4 p.i there were no significance differences in blood chemistry and histopathology between the two groups. However, on day 5 p.i. C227-11*aar*-infected mice had significantly higher BUN (*p* < 0.0001) and higher creatinine values than the wt group (Table 2), a finding indicative of kidney damage in the infected animals. Moderate tubular necrosis of the kidneys (*p* = 0.0114) was observed in the C227-11*aar*-infected group compared to the C227-11-infected group that showed minimal necrosis on day 5 p.i. (Table 2). These greater systemic effects in the C227-11*aar*-infected mice support the hypothesis that the increased levels of Stx2a are responsible for the enhanced morbidity of this mutant, as Stx2a causes renal damage and tubular necrosis in mice intoxicated with Stx2a (Russo et al., 2014). We did not observe significant lesions in cecum or kidneys from mice infected with the s*epA*, *aggA*, or pAA mutant on day 4 (data not shown). However, we noted previously that mice infected with C227-11 exhibit acute tubular renal necrosis at the time of euthanasia (Zangari et al., 2013), so the day 4 time-point was likely too early.

**TABLE 2:** Serum chemistry analysis and histopathology score of four infected C227-11 and C227*aar* mice euthanized on day 4 and 5 post-infection.

	**Day 4 p. i.**			**Day 5 p. i.**		
		
	**Blood chemistry**		**Histopathology score**	**Blood chemistry**		**Histopathology score**
				
**Strain**	**BUN (mg/dl)**	**Creatinine (mg/dl)**	**Kidney**	**BUN (mg/dl)**	**Creatinine (mg/dl)**	**Kidney**
C227-11	32.5	0.185	1.5	25	0.150	1
C227-11*aar*	23.5	0.105	1.5	127.5^a^	0.485	3^b^

*^*a*^*p* < 0.0001 ANOVA Bonferroni’s multiple comparisons test. ^*b*^*p* = 0.0114 ANOVA Uncorrected Fisher’s LSD multiple comparisons test.*

Due to the increased morbidity after infection with C227-11*aar*, additional experiments were done in a C227-11 strain cured of the Stx2a-encoding phage (C227-11ϕcu), so that no toxin would be expressed. We investigated colonization at all intestinal sites and in the feces of mice infected with either C227-11ϕcu or C227-11ϕcu *aar*. Mice were euthanized on either day 1, 3, 5 or 7. We found no difference in colonization between C227-11ϕcu and C227-11ϕcu*aar* at any time points or site (Supplementary Figure S1). These latter data suggest that similarly to the lack of difference between wt and the *aar* mutant in adherence to T84 cells, that mutation of *aar* does not affect colonization by C227-11 in this mouse model.

## Discussion

We sought to elucidate the roles of plasmid-encoded factors in the virulence of the Stx2a + EAEC isolate C227-11 in an Amp-treated mouse model. Although the primary virulence determinant in the Amp-treated mouse model is Stx2a (Zangari et al., 2013), our studies suggest a role for an AggR-regulated factor: the pAA-cured, the *aggR* mutant, and the *aggA* mutant exhibited significantly reduced colonization in the mouse GI tract, and both the pAA-cured and the *aggR* mutant showed reduced morbidity in our model. The critical downstream effector under AggR control is, however, unclear.

Of the >20 AggR-activated genes on the pAA plasmid, all are believed to be involved in some fashion with AAF expression and function. However, a number of AggR-activated chromosomal genes not involved in AAF-mediated adhesion have been described, although their roles in pathogenesis remain obscure. It is possible that one or more chromosomal loci, such as the AggR-regulated AAI type VI secretion cluster, may explain the effects of the pAA and *aggR* mutant phenotypes. Because morbidity in the C227-11-infected Amp-treated model is mostly due to systemic effects of Stx2a, it is possible that the AggR-regulated factor enhances delivery of Stx2a *in vivo*, as we have shown *in vitro* (Boisen et al., 2014).

As we previously reported, Aar acts as an anti-activator protein to repress the effect of AggR and expression of its downstream regulon in the prototype EAEC strain 042 (Santiago et al., 2014). Therefore, we hypothesize that AggR expression is elevated in C227-11*aar*. Consistent with that hypothesis, we found that an *aar* mutant showed increased expression of AAF, and the *aar* mutant caused increased morbidity in Amp-treated mice. We hypothesize that the increased morbidity due to the *aar* mutant is via an effect on expression of an as yet unknown virulence-associated factor under AggR control, given that the *aggA* mutant (lacking the AggA fimbriae) caused *enhanced* weight loss in the Amp-treated mice. It is also possible that there is a factor regulated by Aar, independent of AggR, which is involved in the enhanced virulence of the *aar* mutant. Notably, Santiago et al. (2017) demonstrated, in the EAEC prototype strain 042, that Aar binds directly to H-NS and modulates the H-NS-induced gene expression and, subsequently, mutation of *aar* decreased expression of the H-NS-regulated Lpf fimbriae, LPS-related enzymes, GadXW operon and porins. Alternatively, since both the hyperfimbriated and AggA fimbriae-negative strains exhibit increased morbidity, it is perhaps the dysregulation of fimbriae that affects morbidity, possibly through effects on biofilm formation.

We found that only the C227-11*aggR* mutant was attenuated for lethality in the Amp-treated mouse model, a result that supports the hypothesis that there is an AggR-regulated factor that influences Stx2a delivery in the Amp-treated mice. In contrast, the pAA-cured strain was not attenuated. This latter result suggests that there is a compensatory factor on the pAA plasmid that reduces or delays Stx2a delivery *in vivo*. It may be that the balance between the positive effect on Stx2a delivery by an AggR-regulated factor, and a negative influence from other factors on the pAA plasmid are together responsible for the altered virulence of C227-11 in the Amp-treated mice.

The role of the AAF adhesin remains unclear. A large number of studies implicate this organelle in adherence to human tissue (Harrington et al., 2005; Strauman et al., 2010; Boll et al., 2012, 2013; Boisen et al., 2014; Jønsson et al., 2015), and an AAF pilus gene cluster is found in the large majority of EAEC natural isolates. In contrast, in a recently published rabbit model (Munera et al., 2014), AAF/I did not contribute to virulence of the Stx-EAEC strain studied here. Our data suggest that the presence of an intact AAF increases colonization of the mouse cecum and large intestine, congruent with studies using human tissue. No increased adherence was observed in small bowel sections. As noted above, however, we observed a paradoxical increase in morbidity for the *aggA* mutant in Amp-treated mice. One possible explanation is the fact that our *aggR* mutant displayed greater colonization in the small intestine (though not reaching statistical significance); increased abundance in the small intestine could result in greater Stx delivery. In any case, mouse mortality did not parallel cecal or colonic colonization levels in our studies, suggesting a toxin delivery mechanism distinct from that observed in other Stx-producing *E. coli* infections.

The SPATE proteases of EAEC and *Shigella* spp., including SigA, SepA, and Pic for the C227-11 strain, play various complex roles in pathogenesis, although none have yet been shown to be essential for virulence. Previous reports have suggested that the SPATEs are important for virulence of the Stx-EAEC strain in the rabbit model (Munera et al., 2014) and we showed that SepA from strain C227-11 triggers release of inflammatory cytokines in an *in vitro* T84 cell model (Boisen et al., 2014). However, in the Amp-treated mouse model, SepA was not found to have a role in pathogenesis of strain C227-11.

Overall, we found that the Amp-treated mouse model allowed discernment of a role for one or more AggR-regulated factors in the virulence of the German outbreak isolate C227-11. We hypothesize that AggR-regulated factor(s) allow for greater systemic uptake of Stx2a. The fact that we discovered a role for an EAEC-specific gene in this model which relies largely on the presence of Stx2a suggests that this model may be useful to investigate other putative EAEC virulence factors.

## Data Availability

All datasets generated for this study are included in the manuscript and/or the Supplementary Files.

## Ethics Statement

All animal studies were conducted in accordance with the Guide for the Care and Use of Laboratory Animals (National Research Council, Committee for the Update of the Guide for the Care and Use of Laboratory Animals, 2011) and approved by the Institutional Animal Care and Use Committee of the Uniformed Services University of the Health Sciences and procedures complied with the guidelines in Biosafety in Microbiological and Biomedical Laboratories (Centers for Disease Control and Prevention and National Institutes of Health, 2007).

## Author Contributions

NB conceived, planned, and carried out the experiments with the support of all other authors, and developed the theory and wrote the manuscript with the support of JN and AM-C. TZ and LR provided the experimental support specifically carrying out the animal work. MS carried out the histopathological analysis. A-MH constructed the mutants and provided the analytical support. AO’B contributed to the animal studies by providing the mice, mice facilities, and expertise. JN, FS, and AO’B supervised the project. All authors provided the critical feedback and helped to shape the research, analysis, and manuscript.

## Conflict of Interest Statement

The authors declare that the research was conducted in the absence of any commercial or financial relationships that could be construed as a potential conflict of interest.
