# Supplementary figures and images for: The Role of the AggR Regulon in the Virulence of the Shiga Toxin-Producing Enteroaggregative Escherichia coli Epidemic O104:H4 Strain in Mice
Source: Front Microbiol. 2019 Aug 13;10:1824. doi: 10.3389/fmicb.2019.01824 (PMC6700298; doi:10.3389/fmicb.2019.01824)

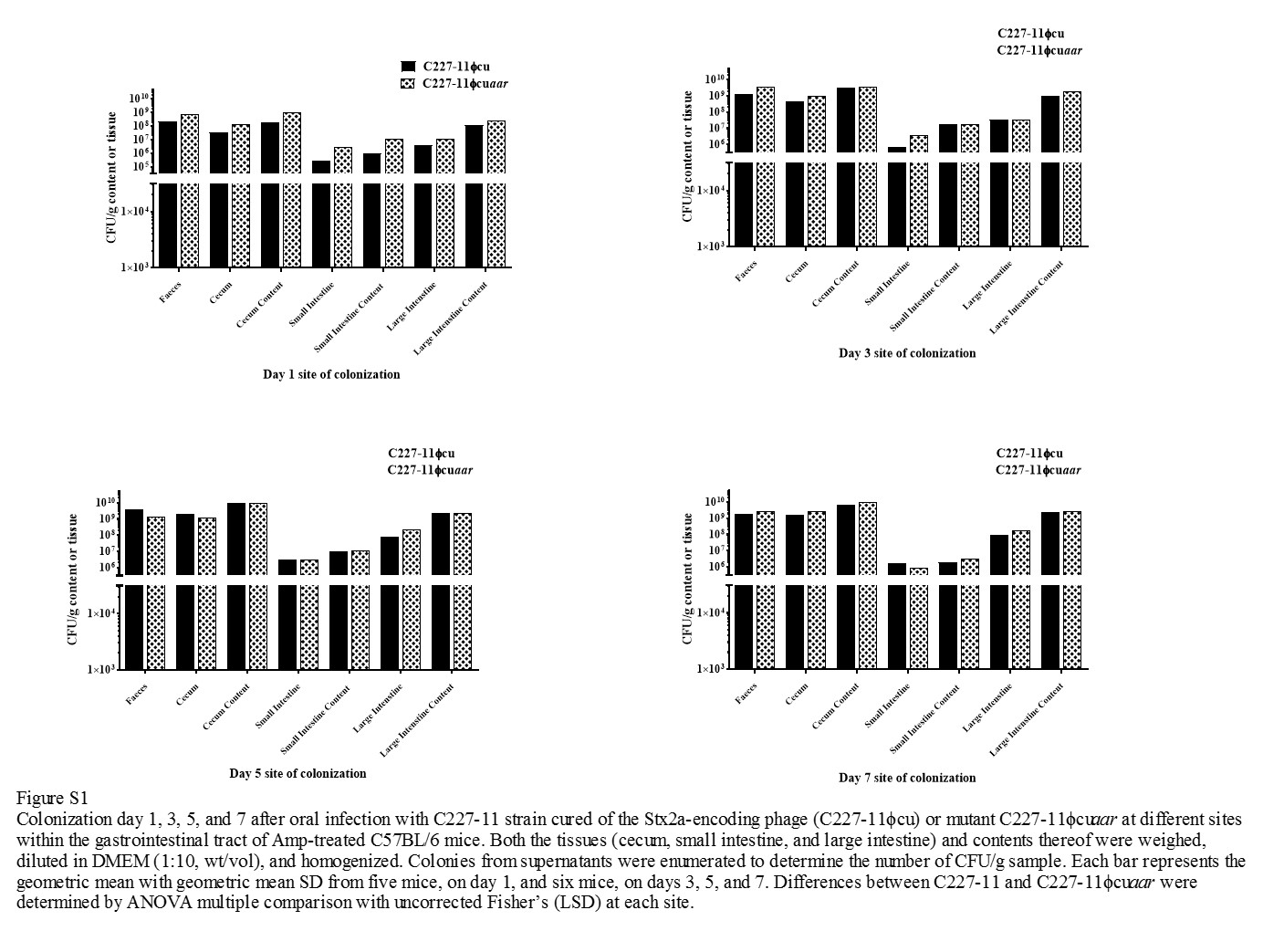

Supplement: Supplementary file 1 [file Image_1.jpg]
